# P-728. Temporal Patterns of Hospitalizations and Clinical Outcomes Associated With Viral Lower Respiratory Tract Disease in 2015–2023: A US Claims Analysis

**DOI:** 10.1093/ofid/ofae631.924

**Published:** 2025-01-29

**Authors:** Susan J Johnson, Ekaterina Maslova, Malin Fageras, Gopal Dalal, Hashmath Ulla T A Syed, Srishti Mishra, Stefan Franzen, Nadir Yehya

**Affiliations:** AstraZeneca, Cambridge, England, United Kingdom; AstraZeneca, Cambridge, England, United Kingdom; AstraZeneca, Cambridge, England, United Kingdom; ZS Associates India Pvt. Ltd., Bengaluru, Karnataka, India; ZS Associates India Pvt. Ltd., Bengaluru, Karnataka, India; ZS Associates India Pvt. Ltd., Bengaluru, Karnataka, India; AstraZeneca, Cambridge, England, United Kingdom; Children’s Hospital of Philadelphia, Philadelphia, Pennsylvania

## Abstract

**Background:**

Although the seasonality of specific respiratory viral infections is well understood, the temporal pattern of severe viral lower respiratory tract disease (LRTD) and its potential consequences have not been well-described. Here we examined the temporal patterns of viral LRTD hospitalizations and associated composite clinical outcomes (invasive mechanical ventilation [IMV]/extracorporeal membrane oxygenation [ECMO] or death) in US clinical practice.

**Figure 1.**
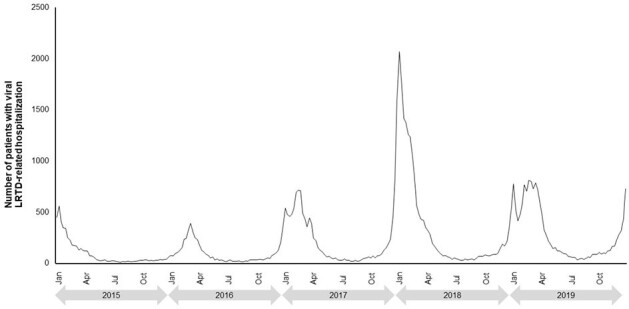
Temporal patterns of viral LRTD hospitalizations from 2015–2019.

**Methods:**

We analyzed data from US adults with a hospitalization and accompanying viral LRTD diagnostic claim (indicated by ICD codes) using Optum’s de-identified Clinformatics® Data Mart Database (2015–2023). IMV/ECMO associated with the hospitalizations was identified through CPT-4, HCPCS and ICD procedure codes. A time-series analysis using raw data was used to construct temporal patterns of viral LRTD-associated hospitalizations, and a composite clinical outcome of IMV/ECMO or death. Data were analyzed separately for 2015–2019 and 2020–2023 due to the impact of COVID-19.

**Figure 2.**
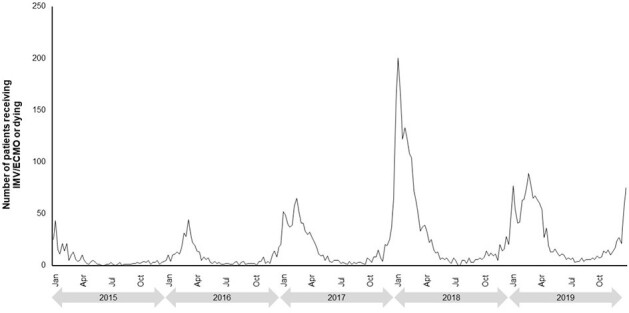
Temporal patterns of IMV/ECMO or death from 2015–2019.

**Results:**

For each year from 2015–2019, peaks generally started towards the end of the calendar year for both viral LRTD hospitalizations (Figure 1) and IMV/ECMO or death (Figure 2). Higher peaks were seen for the 2017–2018 season compared to other years. In 2020–2023 (after the start of the coronavirus pandemic) there were multiple peaks seen throughout each year, with the largest peaks in both hospitalizations (Figure 3) and IMV/ECMO or death (Figure 4) at the end of 2020 and 2021. After the onset of the coronavirus pandemic, the annual peak of IMV/ECMO or death increased relative to the annual peak of hospitalizations (annual peak numbers of IMV/ECMO or death were 7.7–11.2% of the peak number of hospitalizations in 2015–2019, and 14.8–41.6% in 2020–2023). This suggests greater severity of illness and worse mortality among severe viral LRTD in 2020–2023.

**Figure 3.**
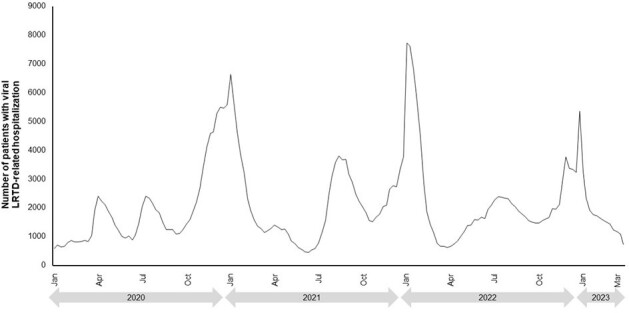
Temporal patterns of viral LRTD hospitalizations from 2020–2023.

**Conclusion:**

Viral LRTD hospitalizations and clinical outcomes place substantial burden on healthcare systems. This considerable variation and unpredictable nature makes healthcare resource planning difficult.

**Figure 4.**
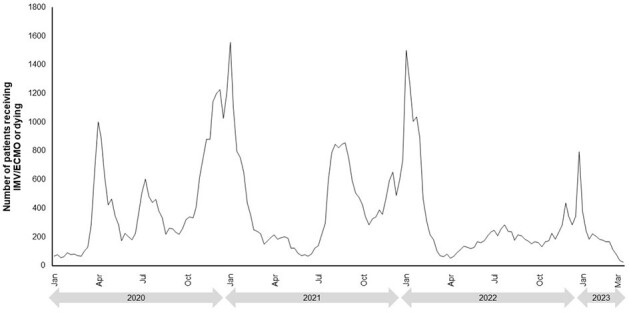
Temporal patterns of IMV/ECMO or death from 2020–2023.

**Disclosures:**

**Susan J. Johnson, PhD**, AstraZeneca: Employee|AstraZeneca: Stocks/Bonds (Public Company) **Ekaterina Maslova, ScD**, AstraZeneca: Employee of AstraZeneca|AstraZeneca: Stocks/Bonds (Private Company) **Malin Fageras, PhD**, AstraZeneca: Full time employee|AstraZeneca: Stocks/Bonds (Private Company) **Gopal Dalal, PhD**, AstraZeneca: Advisor/Consultant|AstraZeneca: Employee of ZS Associates India Pvt. Ltd. and contracted to AstraZeneca at time of study **Hashmath Ulla T. A. Syed, PhD**, AstraZeneca: Advisor/Consultant|AstraZeneca: Employee of ZS Associates India Pvt. Ltd. and contracted to AstraZeneca at time of study **Srishti Mishra, PhD**, AstraZeneca: Advisor/Consultant|AstraZeneca: Employee of ZS Associates India Pvt. Ltd. and contracted to AstraZeneca at time of study **Stefan Franzen, PhD**, AstraZeneca: Stocks/Bonds (Private Company) **Nadir Yehya, MD**, AstraZeneca: Advisor/Consultant

